# Thermal quantitative sensory testing in healthy Dutch children and adolescents standardized test paradigm and Dutch reference values

**DOI:** 10.1186/s12887-017-0827-7

**Published:** 2017-03-16

**Authors:** Gerbrich E. van den Bosch, Monique van Dijk, Dick Tibboel, Abraham J. Valkenburg

**Affiliations:** 1 Intensive Care and department of Pediatric Surgery, Erasmus MC-Sophia Children’s Hospital, Room number SK-3284, PO Box 2060, 3000 CB Rotterdam, The Netherlands; 2 Division of Neonatology, Department of Pediatrics, Erasmus MC-Sophia Children’s Hospital, Rotterdam, The Netherlands

**Keywords:** Children, Pain, Protocol, Quantitative sensory testing, Reference values

## Abstract

**Background:**

Quantitative sensory testing (QST) is often used to measure children’s and adults’ detection- and pain thresholds in a quantitative manner. In children especially the Thermal Sensory Analyzer (TSA-II) is often applied to determine thermal detection and pain thresholds. As comparisons between studies are hampered by the different testing protocols used, we aimed to present a standard protocol and reference values for thermal detection- and pain thresholds in children.

**Methods:**

Our standard testing protocol includes reaction time dependent and independent tests and takes about 14–18 min to complete. Reference values were obtained from a sample of 69 healthy term born children and adolescents with a median age of 11.2 years (range 8.2 to 17.9 years old). Seventy-one children were recruited and data of 28 males and 41 females was obtained correctly. We studied possible age and sex differences.

**Results:**

This study provides Dutch reference values and presents a standard quantitative sensory testing protocol for children with an age from 8 years onwards. This protocol appeared to be feasible, since only two out of 71 participants were not able to correctly complete the protocol due to attention deficits and were therefore excluded. We found some significant age and sex differences: females were statistically significantly more sensitive for both cold and heat pain compared to males, and the youngest children (8–9 years old) were less sensitive to detect a warm stimulus. The youngest children tend to be more sensitive to heat pain in comparison to older participants, although the difference was not statistically significant.

**Conclusions:**

We present a feasible thermal quantitative sensory testing protocol for children and reference values that are easy to interpret and may serve as normative values for future studies.

**Electronic supplementary material:**

The online version of this article (doi:10.1186/s12887-017-0827-7) contains supplementary material, which is available to authorized users.

## Background

Quantitative Sensory Testing (QST) encompasses a group of assessments with the goal to systematically document the functioning of the sensory nervous system, and in particular, the nociceptive system. The advantage of QST in comparison with a classical neurological examination is its quantitative nature. Furthermore, depending on the type of stimuli, both large myelinated and small myelinated nerve fibers in combination with unmyelinated nerve fibers can be tested, because QST can involve thermal, pressure, vibration or electrical stimulation [1]. QST is widely used in adults to diagnose and monitor neuropathic and chronic pain disorders [2]. Therefore, the German research network on neuropathic pain (DFNS) developed a standard, comprehensive testing protocol [3].

The first use of QST in children with regards to the diagnosis and monitoring of pain syndromes was reported in 1987 for the diagnosis of diabetic complications [4]. Since then, many different devices to determine pain thresholds, pain intensity, and pain tolerance have been tested in children, for example the Cold Pressor Task [5], the VibraMeter [6] and the Thermal Sensory Analyzer [7]. The German protocol has also been evaluated for the ability to diagnose chronic pain in children, and reference values for several different tests are available [7]. Those reference values showed that 6–8 year old children were in general less sensitive to detect a thermal or mechanical stimulus compared to older 9–12 year old children. On the other hand, the younger children were more sensitive to pain stimuli compared to the older children. Furthermore, girls appeared to be more sensitive to thermal detection [8] and pain stimuli compared to boys [7, 9].

Besides the diagnosis of chronic and neuropathic pain, QST is used for basic mechanistic studies of pain as a neurobiological phenomenon in healthy volunteers, as well as in pharmacological studies evaluating the efficacy of analgesics [2]. QST is also an often-used technique for experimental pain research in children. Especially by using a thermal stimulation paradigm, detection- and pain thresholds can easily be determined in children. The assessment of thermal detection thresholds is feasible in children from the age of 5 years onwards [10]. The Thermal Sensory Analyzer (Medoc Ltd. Advanced Medical Systems, Ramat Yishai, Israel), for example, is previously used to investigate the long-term effects of neonatal pain and analgesic treatments in children. Hermann and colleagues showed that former preterm (*n* = 19) and term born (*n* = 20) patients with a history of neonatal intensive care unit (NICU) admission were less sensitive for brief heat pain stimuli than controls (*n* = 20) [11]. In a larger study by Walker and colleagues, former extremely preterm NICU patients (*n* = 43) appeared to be less sensitive for the detection of cold and warmth stimuli and had higher cold and heat pain thresholds compared to controls (*n* = 44) [12]. In each study, subjects were compared with healthy controls. However, comparison between different studies is hampered by the lack of uniform testing protocols and reference values. Some studies measured a thermal threshold for actual pain [13], while others measured a thermal threshold for unpleasantness rather than for pain [7]. Therefore, the aim of the present study is to provide reference values for 8–17-year-old children and adolescents and to present a standard thermal QST testing protocol which is not time consuming and useful for repeated evaluation over time.

## Methods

### Participants

Participants were recruited as healthy controls for a neuroimaging study regarding the long-term effects of early pain [14]. Besides Magnetic Resonance Imaging (MRI) scans, thermal QST tests were performed and the results are used for this current study. These QST data were available and represent a sample of the Dutch population.

The healthy subjects were recruited through two different mechanisms. First, all included participants were asked whether they could recommend someone else in the age range of 8–18 years who would also be interested in volunteering. Potential candidates were sent an invitation letter and were contacted 2 weeks later by phone to ask if they were interested in participation. Invitations were also sent to parents of children of three primary schools in Rotterdam. Parents were asked to contact the researcher to make an appointment for the study. Only term born children and adolescents aged 8 years up to and including 17 years old were included. Exclusion criteria were the following: a history of severe early pain such as surgery in the neonatal period, preterm birth, intellectual disabilities, or gross motor or sensory disabilities.

This study was performed at the Erasmus University Medical Center (Erasmus MC) in Rotterdam in compliance with the Code of Ethics of the World Medical Association (Declaration of Helsinki) and was approved by the Institutional Review Board of Erasmus MC. Informed consent was obtained from the parents of each subject prior to participation. According to Dutch law informed assent was also obtained from children 12 years of age and older prior to participation. Recruitment into the study took place from June 2011 to March 2013.

### Materials

**Fig. 1:**
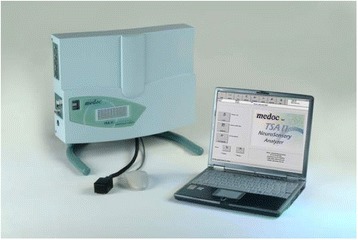
Thermal Sensory Analyzer-II (Medoc Ltd. Advanced Medical Systems, Ramat Yishai, Israel)

**Fig. 2:**
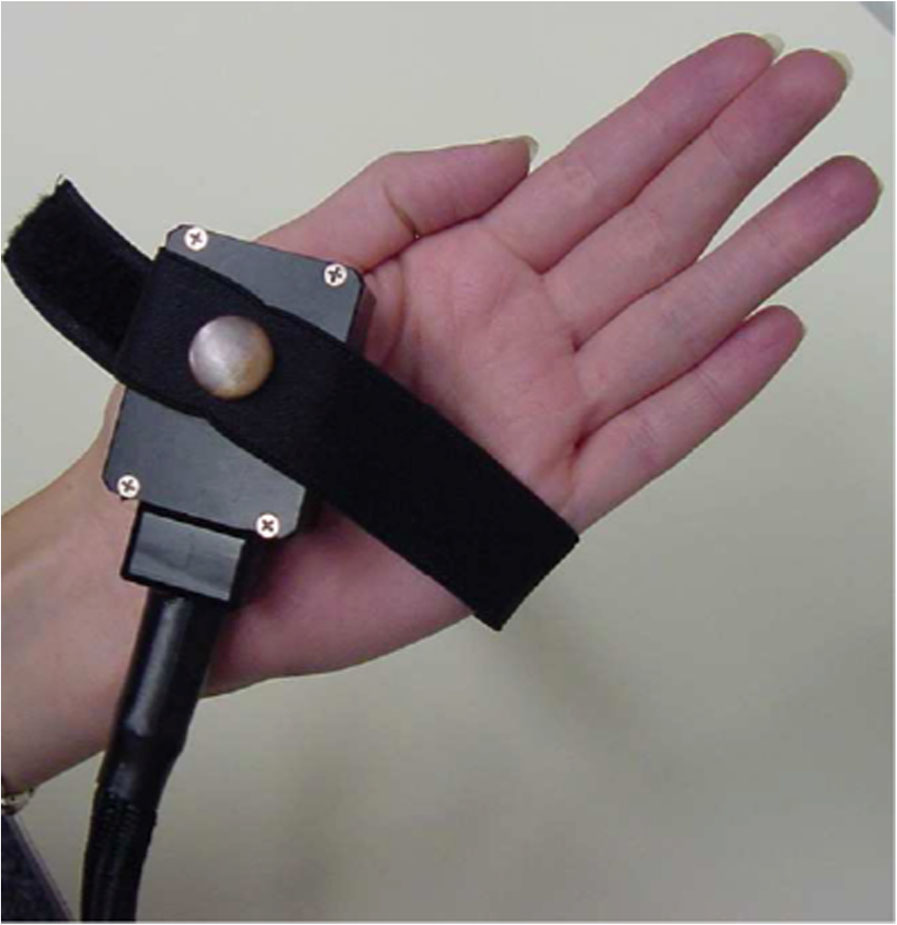
Peltier-based contact thermode (30 × 30 mm)

QST tests were performed with the computer-controlled Thermal Sensory Analyzer (TSA type II, Medoc Ltd. Advanced Medical Systems, Ramat Yishai, Israel) (Fig. 1) with a Peltier-based contact thermode (30 × 30 mm) (Fig. 2). WinTSA software (version 5.35) served to determine the detection- and pain thresholds, and a subtest of the Amsterdam Neuropsychological Tasks (ANT) [15] was used to measure visual-motor reaction time.

### Test protocol

In previous QST studies at our department we used the same standardized TSA-II test protocol to determine detection- and pain thresholds [10, 16]. The protocol is structured as follows: explaining the procedure to the subject in less than a minute, determining visual-motor reaction time since one of the QST subtests is reaction time dependent (2–3 min) [15], determining detection- and pain thresholds using the reaction time dependent Method of Limits (MLI) (8–10 min), and determining detection thresholds using the reaction time independent Method of Levels (MLE) (4–5 min). Thus, the entire protocol takes approximately 14–18 min. The entire TSA-II thermode-stimulating surface was placed in contact with the skin of the thenar eminence of the non-dominant hand and was firmly secured by a Velcro band. The non-dominant hand was chosen so as to allow the subject to use the dominant hand for clicking the button during the MLI subtest. Detection thresholds were measured with two methods, MLI and MLE, as these are both commonly used in the literature [7, 10–12, 16, 17]. Furthermore, a previous study in 5-year-old children demonstrated significant differences between both methods in which the MLE established more sensitive detection thresholds compared to the MLI [10]. Another study in 6 to 17-year-old subjects also found more sensitive detection thresholds using the MLE compared to the MLI technique [17]. All QST tests in this study were conducted by the same researcher (GB).

### Preparation

Skin temperature of the thenar eminence was measured with a skin thermometer. Room temperature was measured to ensure that the test environment was the same for every subject. After this, the protocol was explained to the child and his or her parents. It was emphasized that testing could not harm the hand, and parents were asked not to interact with their child during the assessment.

### Visual-motor reaction time

After preparation, the child’s reaction time was determined with the short *base-line speed task* of the Amsterdam Neuropsychological Tasks (ANT) [15]. In case of differences in reaction time between groups, it is possible to correct for reaction time in the MLI group analysis.

### MLI

Next, detection thresholds for cold and warmth were determined using the MLI technique. The baseline temperature of the thermode was set at the standard temperature of 32 °C (centre of neutral range). From baseline, the temperature was steadily lowered at a rate of 1 °C/s. The researcher instructed the participant as follows: “The thermode is going to become cold, press the button as soon as you feel the temperature changing”. After the button was pressed, the temperature returned to 32 °C at a rate of 1.0 °C/s. This was repeated five times with 6 s between each stimulus. The first two stimuli served as rehearsal stimuli. The detection threshold was calculated as the mean value of the last four temperatures. Next, the temperature was steadily increased at a rate of 1 °C/s to determine the detection threshold for warmth using the same technique.

Subsequently, the MLI technique was applied to determine pain thresholds for cold and heat. Starting again from the baseline temperature of 32 °C, the temperature was steadily lowered at a rate of 1.5 °C/s. The researcher instructed the participant as follows: “The thermode is going to become cold, press the button as soon as the thermode starts to feel painful”. After the button was pressed, the temperature returned to 32 °C at a rate of 10.0 °C/s. This was repeated four times with 10 s between each stimulus. The first stimulus served as a rehearsal stimulus and the cold pain threshold was calculated as the mean value of the last four temperatures. We used one test stimulus instead of two since the children were already familiar with the test and material due to the foregoing determination of the detection thresholds. Next, the pain threshold for heat was determined in the same manner. When the child did not press the button before the minimum temperature of 0 °C or the maximum temperature of 50 °C, the test automatically terminated. In that case, the cut-off temperature of 0 °C or 50 °C was used in the calculation of the mean threshold and the fact that the participant did not reach his or her pain threshold was made note of.

### MLE

Next, detection thresholds for cold and warmth were determined with the MLE technique to obtain thresholds without the possible influence of reaction time. The researcher told the child that the thermode would either become colder, or would not change in temperature. The first thermal stimulus was 3.0 °C below the baseline temperature of 32.0 °C. Following each thermal stimulus the researcher asked “Did the thermode become cold or not?” The researcher pressed the ‘yes’ or ‘no’ button of the mouse depending on the answer. If the participant experienced difference in temperature of the thermode, the target temperature would decrease and if the participant did experience a cold sensation, the target temperature would became less cold. The temperature step size was halved every time the participant experienced cold; the next stimulus decreased with half of the previous step size from baseline. In case the child did not experienced cold, the temperature decreased with the same step size estimated from the prior temperature. The test terminated when the step size had decreased to a level of 0.1 °C and this temperature was registered as the detection threshold by the TSA-II. The number of stimuli needed to decrease the step size to 0.1 °C was registered as well. The warm detection threshold was determined in the same manner starting with a stimulus temperature of 3.0 °C above the baseline temperature. Pain thresholds were not measured with the MLE technique since temperatures above the pain threshold are reached which is unfavourable in children.

### Statistical analysis

Only test results of children who correctly finished the test protocol (without attention deficits throughout the test) were included in the statistical analyses. Normally distributed variables are presented as mean (standard deviation) and non-normally distributed variables as median (interquartile range). The detection- and pain thresholds are presented as both mean and median values. We defined four age groups: 8–9 years, 10–11 years, 12–13 years, and 14–17 years old. Differences in demographic characteristics between those age groups and between sexes were determined with independent samples *t*-test for two groups or ANOVA for more than two groups (with post hoc Bonferroni correction) for continuous data and chi square tests for categorical data. Detection thresholds obtained by the MLI and MLE, and pain thresholds obtained by the MLI were compared between age groups and sexes using an independent samples *t*-test or ANOVA (with post hoc Bonferroni correction). Additionally, linear regression analyses (which are in essence the same as ANCOVA tests but nowadays more often applied) served to correct for the mean reaction time. Numbers of children who did not reach a pain threshold during the MLI were compared between groups using a chi square test. Correlations between detection thresholds obtained with the MLI and the MLE, and between reaction time and thresholds obtained with the MLI, were determined using Pearson product moment correlation coefficients. A *p*-value of 0.05 or less was considered statistically significant. Analyses were conducted using SPSS 20.0.

## Results

### Demographic data

Seventy-five eligible subjects were recruited. Two children (8 and 9 years old) who were not able to correctly conduct the test due to attention deficits were excluded. One of them had already been diagnosed with attention deficit hyperactivity disorder (ADHD) prior to the study. Furthermore, four children were preterm born and were therefore excluded from the analyses afterwards. All the 69 remaining subjects correctly completed the entire QST test without attention deficits in approximately 14–18 min (including explanation). The subjects were aged 8 to 17 years with a median age of 11.2 years (IQR 10.2 to 12.6 years). Twenty-eight were males (40.6%; Table 1). Demographic characteristics per age group are presented in Table 1. Moreover, skin temperature (mean 36.7° Celsius (SD 0.9)) and room temperature (mean 23.0° Celsius (SD 1.3)) did not significantly differ between the age groups (*p* = 0.72 and *p* = 0.47, respectively). Reaction time differed significantly between age groups (*p* = 0.02; post-hoc Bonferroni correction: 10–11 year versus 14–17 years; *p* = 0.02), indicating a faster reaction time in the oldest subjects. These values are presented in Table 1. There were no statistically significant differences in age, skin temperature, room temperature, or reaction time between males and females.

**Table 1:** Demographic characteristics

Control group	Total group	8–9 years	10–11 years	12–13 years	14–17 years
(*n* = 69)	(*n* = 69)	(*n* = 14)	(*n* = 31)	(*n* = 12)	(*n* = 12)
Age Years, Median (IQR)	11.2 (10.2 to 12.6)	9.0 (8.7 to 9.4)	11.1 (10.6 to 11.3)	12.5 (12.5 to 13.0)	16.5 (14.7 to 17.6)
Sex n (%) Male	28 (40.6)	6 (42.9)	13 (41.9)	4 (33.3)	5 (41.7)
Ethnicity n (%) Western European	47 (68.1)	7 (50.0)	20 (64.5)	9 (75.0)	11 (91.7)
Handedness n (%) Right	66 (95.7)	13 (92.9)	31 (100)	11 (91.7)	11 (91.7)
Reaction time ms, Median (IQR)	297 (274 to 327)	313 (290 to 335)	307 (280 to 357)	300 (260 to 310)	259 (238 to 294)

**Table 2:** Detection- and pain thresholds per age group

Control group		Total group	8–9 years	10–11 years	12–13 years	14–17 years	*P*-value
(*n* = 69)		(*n* = 69)	(*n* = 14)	(*n* = 31)	(*n* = 12)	(*n* = 12)	
Method of Limits (MLI)							
Cold detection threshold °C	Mean (SD)	30.7 (0.7)	30.6 (0.9)	30.6 (0.8)	30.8 (0.5)	31.0 (0.4)	0.43
	Median (IQR)	30.9 (30.4 to 31.1)	30.8 (30.3 to 31.1)	30.8 (30.3 to 31.1)	30.8 (30.6 to 31.1)	31.0 (30.8 to 31.3)	
Warm detection threshold °C	Mean (SD)	33.9 (1.2)	34.6 (1.7)	33.8 (0.9)	34.1 (1.1)	33.2 (0.5)	0.01*
	Median (IQR)	33.5 (33.1 to 34.3)	34.1 (33.5 to 35.9)	33.5 (33.2 to 34.0)	33.9 (33.3 to 35.0)	33.1 (32.9 to 33.2)	
Cold pain threshold °C	Mean (SD)	10.0 (9.1)	9.7 (10.8)	9.2 (9.4)	12.3 (9.0)	10.0 (6.7)	0.81
	Median (IQR)	8.0 (0.7 to 17.7)	3.9 (0.0 to 21.8)	7.3 (0.0 to 15.1)	14.6 (3.0 to 19.1)	11.3 (3.1 to 15.3)	
Threshold not reached	N (%)	27 (39)	8 (57.1)	14 (45.2)	3 (25.0)	2 (16.7)	0.12
Heat pain threshold °C	Mean (SD)	45.9 (4.2)	43.2 (5.4)	46.9 (3.7)	45.9 (4.0)	46.2 (3.2)	0.051**
	Median (IQR)	47.2 (42.2 to 50.0)	41.7 (38.5 to 49.3)	47.7 (44.6 to 50.0)	46.9 (41.8 to 50.0)	47.1 (43.2 to 49.3)	
Threshold not reached	N (%)	28 (41)	6 (42.9)	16 (51.6)	4 (33.3)	2 (16.7)	0.20
Method of Levels (MLE)							
Cold detection threshold °C	Mean (SD)	30.8 (1.2)	30.5 (1.4)	30.6 (1.4)	31.0 (0.6)	31.2 (0.4)	0.29
	Median (IQR)	31.2 (30.4 to 31.5)	31.0 (29.9 to 31.5)	31.2 (30.4 to 31.5)	31.3 (30.4 to 31.5)	31.4 (31.2 to 31.5)	
Number of stimuli	Mean (SD)	11 (3)	11 (4)	11 (3)	10 (3)	12 (3)	0.24
	Median (IQR)	10 (9 to 12)	11 (9 to 13)	11 (9 to 12)	9 (8 to 11)	12 (9 to 14)	
Warm detection threshold °C	Mean (SD)	33.6 (1.0)	33.7 (1.1)	33.7 (0.9)	33.6 (1.2)	33.1 (0.7)	0.21
	Median (IQR)	33.6 (32.9 to 34.1)	33.4 (32.8 to 34.4)	33.9 (33.1 to 34.1)	33.4 (32.6 to 34.0)	32.8 (32.4 to 33.9)	
Number of stimuli	Mean (SD)	9 (3)	10 (3)	9 (3)	9 (2)	10 (2)	0.25
	Median (IQR)	9 (7 to 11)	10 (8 to 12)	8 (7 to 11)	8 (7 to 10)	10 (9 to 12)	

ANOVA test for continuous data and Chi squared test for categorical data were used to test differences between the four age groups

*Post-hoc Bonferroni correction: 8–9 year old versus 14–17 years old; *p* = 0.01

**Post-hoc Bonferroni correction: 8–9 year old versus 10–11 years old; *p* = 0.04

### QST reference data

#### Total group MLI and MLE

**Fig. 3:**
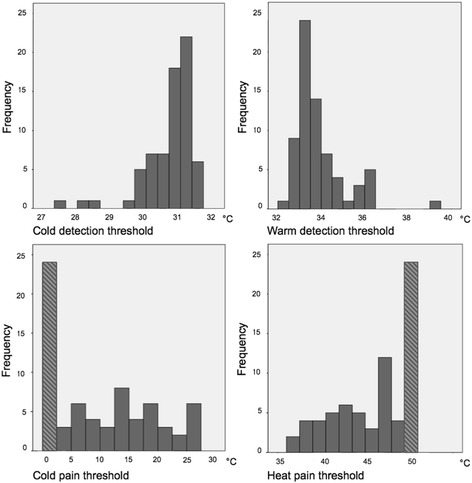
Histograms of the total group *N* = 69, Method of Limits.  These bars include subjects who did not reach their pain threshold before the minimum/maximum temperature was reached. The cut-off temperature of 0 °C or 50 °C was used in the calculation of the mean threshold

Mean values and standard deviations of the detection- and pain thresholds are presented in the left-hand column of Table 2. Regarding the pain thresholds for cold and warmth, around 40% of the participants did not reach their pain threshold at least one time during the test (out of the four stimuli). The detection thresholds obtained with the MLI were highly correlated to the detection thresholds obtained with the MLE (*p* < 0.001, correlation coefficients MLI cold and MLE cold 0.63, MLI warmth and MLE warmth 0.52). The reaction time obtained with the ANT was not correlated to the four MLI modalities (detection threshold cold: *p* = 0.16, detection threshold warm: *p* = 0.12, pain threshold cold: *p* = 0.28, and pain threshold heat: *p* = 0.94 with correlation coefficients of respectively − 0.17, 0.19, 0.13 and 0.01). Histograms with the thresholds of the total group obtained with the MLI are presented in Fig. 3.

#### Age effects

Age effects were found in the warm detection threshold obtained with the MLI, indicating a higher detection threshold for warmth in the youngest children (34.6 SD 1.7) compared to the oldest group (33.2 SD 0.5) (*p* = 0.01). No significant differences were found in the detection threshold for warmth obtained with the MLE, and in detection thresholds for cold obtained with both the MLI of the MLE technique. Furthermore, with regards to the heat pain threshold a lower threshold in the age group 8–9 years was found (43.2 SD 5.4) compared to age group 10–11 years, although the difference was not statistically significant (46.9 SD 3.7; *p* = 0.051, Table 2). After additional correction for the mean reaction time, results remained comparable (warm detection threshold *p* = 0.02; heat pain threshold *p* = 0.053). Histograms with the thresholds per age group obtained with the MLI are presented in Additional file 1: Figure S4a-d.

#### Sex effects

No statistical significant differences in detection thresholds obtained with both the MLI and the MLE technique were found between males and females. Regarding pain thresholds, females were statistically significantly more sensitive for both cold (females 12.0 SD 9.4, males 7.0 SD 7.9; *p* = 0.03) and heat pain (females 44.9 SD 4.3, males 47.3 SD 3.7; *p* = 0.02) compared to males. Furthermore, more than twice as many males did not reach their pain threshold for cold (males 57.1%, females 26.8; *p* = 0.01) and for heat (males 60.7%, females 26.8; *p* = 0.01), compared to females. Histograms with the separate thresholds of females and males obtained with the MLI are presented in Additional file 1: Figure S4a-d.

## Discussion

The aim of this study was to provide Dutch reference values and a standardized testing protocol for thermal quantitative sensory testing in children and adolescents. Through the years, we have gained much experience with this testing protocol and noticed that it is very easy to conduct in children [10, 16]. In this current study we obtained correctly obtained QST data from almost all participants. Only two subjects could not complete the protocol correctly due to attention deficits. One of them was already diagnosed with ADHD. Furthermore, the testing protocol is not time consuming since it only takes 14–18 min to complete.

Two other studies have provided protocols and reference values for thermal quantitative sensory testing in children with the use of the TSA-II [7, 17]. The differences between their protocols and ours are summarized in Table 3. In general, the protocol of Meier and colleagues (2001) is comparable to our protocol. However, they do not specify when the child had to press the button during the determination of the pain thresholds and state that the quality of thermal pain perception (burning versus pricking etcetera) was not assessed [17]. Furthermore, sex- or age differences were not described and individual reaction time was not assessed in that study. Valid comparison with our reference values is not possible. Yet, the detection thresholds obtained with the MLI are roughly the same, while the pain thresholds differ more than 4 °C, suggesting a higher sensitivity for both cold and heat pain in the study by Meier and colleagues [17]. However, these differences in reference values could have been caused by different instructions given to the subjects rather than actual differences in pain sensitivity between children in both studies, since we do not know which instructions were given in this previous study. In the recent study by Blankenburg and colleagues, children were instructed to press the button of the TSA-II as soon as the thermode started to sting, ache or burn [7]. In our study children were asked to press the button during the MLI pain subtests as soon as the temperature started to feel painful. We preferred to use the word ‘painful’ rather than other explicit words such as ‘aching’ or ‘burning’. Pain is a subjective experience and therefore we decided not to describe it with other words than ‘painful’. Therefore our reference values represent actual pain thresholds. This may probably explain why our values are much higher than in the study by Blankenburg and colleagues (6 °C or more difference for cold pain and 2 or more for heat pain depending on age and sex) [7]. The fact that Blankenburg and colleagues measured thresholds on the dorsal side of the hand instead of the thenar eminence could also have been a reason for differences between their study and ours. Furthermore, Blankenburg and colleagues used a logarithmic data transformation for their detection thresholds since the data were not normally distributed, which distorts comparison to our reference values. Many previous clinical studies in children did not present logarithmic transformed data, in line with our study.

**Table 3:** Comparison between different protocols and reference values in children using the TSA-II

	Meier et al. 2001	Blankenburg et al. 2010	Van den Bosch et al. 2017
Sample size	*N* = 101	*N* = 176	*N* = 69
Sex differences	Not tested *(53 girls and 48 boys were included)*	88 males versus 88 females	28 males versus 41 females
Age differences	Not tested *(Included children were 6–17 years old)*	3 different age groups; Age range 6–16 years	4 different age groups; Age range 8–17 years
QST technique	Thermal detection and pain and vibration sensation	Thermal and mechanical detection and pain	Thermal detection and pain
Instructions for pain threshold	Not specified	‘Aching’, ‘stinging’, or ‘burning’	‘Starts to feel painful’
Reaction time	Not obtained	Not obtained	Obtained
Data transformation	None	Logarithmic data transformation	None
Body site	Hand and foot	Face, hand and foot	Hand
Time	Not described for the total protocol	32.0 ± 3.5 min in adolescents (13–16 years) and 35.0 ± 6.2 min in children (9–12 years)	14–18 min

We found only small age effects with respect to the detection threshold for warmth and the pain threshold for heat measured with the MLI, in which the youngest children were less sensitive to detect a warm stimulus but – interestingly – more sensitive to heat pain in comparison to older participants, although this last finding was not statistically different. Our results are in line with a previous study that found that 6 to 8-year-old children (24 boys and 24 girls) were generally less sensitive to thermal and mechanical detection stimuli but more sensitive to all pain stimuli than 9 to 12-year-old children (32 boys and 32 girls), whereas the differences between these older children and adolescents (13–17 years; 32 boys and 32 girls) were slight [7]. However, neither the detection thresholds obtained with the MLE nor detection and pain thresholds for cold differed between our age groups. Although reaction time was not significantly correlated to the MLI thresholds, differences in attention among age groups during the MLI tests could possibly have influenced the results. Reaction time was measured at the start of the test protocol when the attention of the subject was probable the highest. Since attention deficits have less influence on MLE results, this could explain the absence of age group differences using the MLE technique. Moreover, the variance in pain thresholds for heat is smaller in comparison with the variance for cold pain thresholds, therefore significant differences between age groups are easier to detect with respect to heat pain thresholds.

Furthermore, girls proved more sensitive than boys to both cold and heat pain stimuli. This is also in line with other studies and a meta-analysis [7, 9]. Therefore we recommend matching on gender. Additionally, boys statistically significantly reached their pain threshold for both cold and heat less often than girls. A previous version of the TSA permitted to lower the minimum temperature of the TSA-II to − 10 °C, instead of 0 °C. This can be a solution to avoid participants not reaching their pain threshold for cold, however the question arises whether this is ethical justifiable for studies in children. Moreover, we recommend measuring every participant’s reaction time even though in the present study it was not significantly correlated to the reaction time dependent MLI subtests. It is a short test, which takes 2 min to conduct, and in case there are differences between groups with respect to reaction time it is possible to correct for it. In a previous study of our research group in younger children, however, the detection thresholds obtained in a reaction time dependent fashion were significantly correlated to IQ, while the detection thresholds obtained in a reaction time independent fashion were not [10]. Unfortunately reaction time was not tested in this previous study [10].

We chose to measure the detection- and pain thresholds with thermal stimuli using the TSA-II because it is feasible and therefore often used in experimental pain research in children [10–12]. Since the device is MRI compatible, it also gains popularity in functional MRI studies measuring brain activation during pain (other reports of our department involving MRI in combination with thermal stimuli are forthcoming) [13, 18]. To be able to compare our results with previous studies, we chose to obtain detection- and pain thresholds with the TSA-II as well. However, a few features speak against its use: it is an expensive device, and instructions need to be standard and unambiguous to avoid that one child during the MLI pain test will press the button when the temperature starts to hurt and another when it starts to itch for example. Future studies that will test the inter-instructor variability would be valuable.

Possible alternatives are techniques using cold water or electrical stimuli, which are also often used in children. A popular test to determine pain intensity and tolerance is the cold pressor task [5, 19, 20] in which children immerse a hand or forearm in cold water and give pain scores for the duration of the test. These scores are thought to reflect the pain intensity experienced. Furthermore, the immersion time gives information about pain tolerance [19, 20]. The cold pressor task has several complexities such as including variations in the circulation and the turbulence of the cold water. The Neurometer (Neurotron, Inc., Baltimore, MD, USA) allows for electrodiagnostic sensory nerve testing [21] but is very painful and will therefore probably frighten children. Furthermore, it is less used in previous studies compared to the other techniques mentioned above.

Our standardized protocol only takes 14–18 min to complete and is therefore also useful in clinical practice for diagnostic purposes [16]. In a child with congenital pain insensitivity syndrome we found elevated detection- and pain thresholds measured with both the MLI and MLE technique [16]. The TSA-II is also used for the detection of neuropathies in adults [22]. This study found that the TSA-II had a sensitivity of 72% for the diagnosis of small fiber neuropathy and authors recommended the measurement of both cold and warmth detection thresholds [22]. Our protocol includes both the MLI and the MLE technique and since the results cannot be used interchangeably we prefer to include both tests in the protocol. However, since MLI and MLE findings are highly correlated, the test protocol could be shortened by only using the MLI technique for both the determination of the detection- and pain thresholds in children from 8 years onwards instead of using the MLE technique. Since the MLI technique is preferred for the determination of pain thresholds in children, we advise to use the MLI also for the determination of the detection thresholds in order to be consistent in all the different modalities, even though the MLE technique appears to be a bit more sensitive for the determination of detection thresholds in children [10, 17]. In adults MLE is used for the determination of pain thresholds [13], but the disadvantage is that it is more time-consuming than the MLI pain test and that temperatures above the pain threshold are reached. For specific groups such as for younger children, however, the MLE technique is preferred rather than the MLI technique with respect to detection threshold measurements [10].

The strength of our reference values is that they are easy to interpret and may serve as normative values for future studies. The sample size was relatively small when compared to the studies of Blankenburg et al. and Meier et al. [7, 17], however, our sample is larger than control groups in previous studies [10, 11, 16]. A severe limitation of our study is that the age groups in our study did not have balanced numbers of children per group. We chose for small intervals in age per group rather than balanced sample sizes per age group. Moreover, it could be that our sample is biased towards children with a higher pain tolerance since children with fear for pain might not have volunteered in our study. On the other hand, the participants did not undergo this QST test before and were therefore not aware of the type and intensity of the painful stimulus that they would receive. Another limitation is that the minimum/maximum temperature of 0 °C or 50 °C was used in the calculation of the mean threshold in case the participant did not reach his or her pain threshold. Unfortunately 39% of our participants did not reach their pain threshold for cold and 41% did not reach their pain threshold for heat. The chosen values might have distorted the mean and standard deviation of the pain threshold values. However, every other arbitrary chosen value would have potentially influenced the results as well. A solution could be to exclude subjects who do not reach their threshold. However, this has the major disadvantage that the mean threshold of the group would then be biased towards a lower pain threshold. Moreover, possible habituation could have occurred during the measurement of the pain thresholds. However, we have used the mean value out of four stimuli to encounter this possible mechanism [23]. Other possible limitations are the testing at only one body site and the application of thermal quantitative sensory testing only. However, the positive side is that this design enabled us to complete the entire protocol in no more than 14–18 min, which decreases the risk for fatigue and distraction in children.

## Conclusion

We conclude that this study protocol is applicable for children from 8 years onwards, not time consuming and feasible even for daily practice. Furthermore, we provide easy interpretable thermal detection and pain reference values for 8 to 17-year-old children and adolescents. Our study has the advantages that we have included both the MLI and MLE technique, present reference values for reaction time and present actual pain thresholds rather than unpleasantness thresholds. Hopefully future studies will use our study protocol as well and will present the outcomes of their cohorts.

## Additional file

Additional file 1: Figure S4
**a** - Cold detection threshold. Histograms per subgroup. **b** - Warm detection threshold. Histograms per subgroup. **c** - Cold pain threshold. Histograms per subgroup. **d** - Heat pain threshold. Histograms per subgroup. (ZIP 329 kb)

## Abbreviations

ANT: Amsterdam Neuropsychological Tasks
MLE: Method of Levels
MLI: Method of Limits
QST: Quantitative Sensory Testing
TSA: Thermal Sensory Analyzer
