# Supplementary figures and images for: Thermal quantitative sensory testing in healthy Dutch children and adolescents standardized test paradigm and Dutch reference values
Source: BMC Pediatr. 2017 Mar 16;17:77. doi: 10.1186/s12887-017-0827-7 (PMC5356312; doi:10.1186/s12887-017-0827-7)

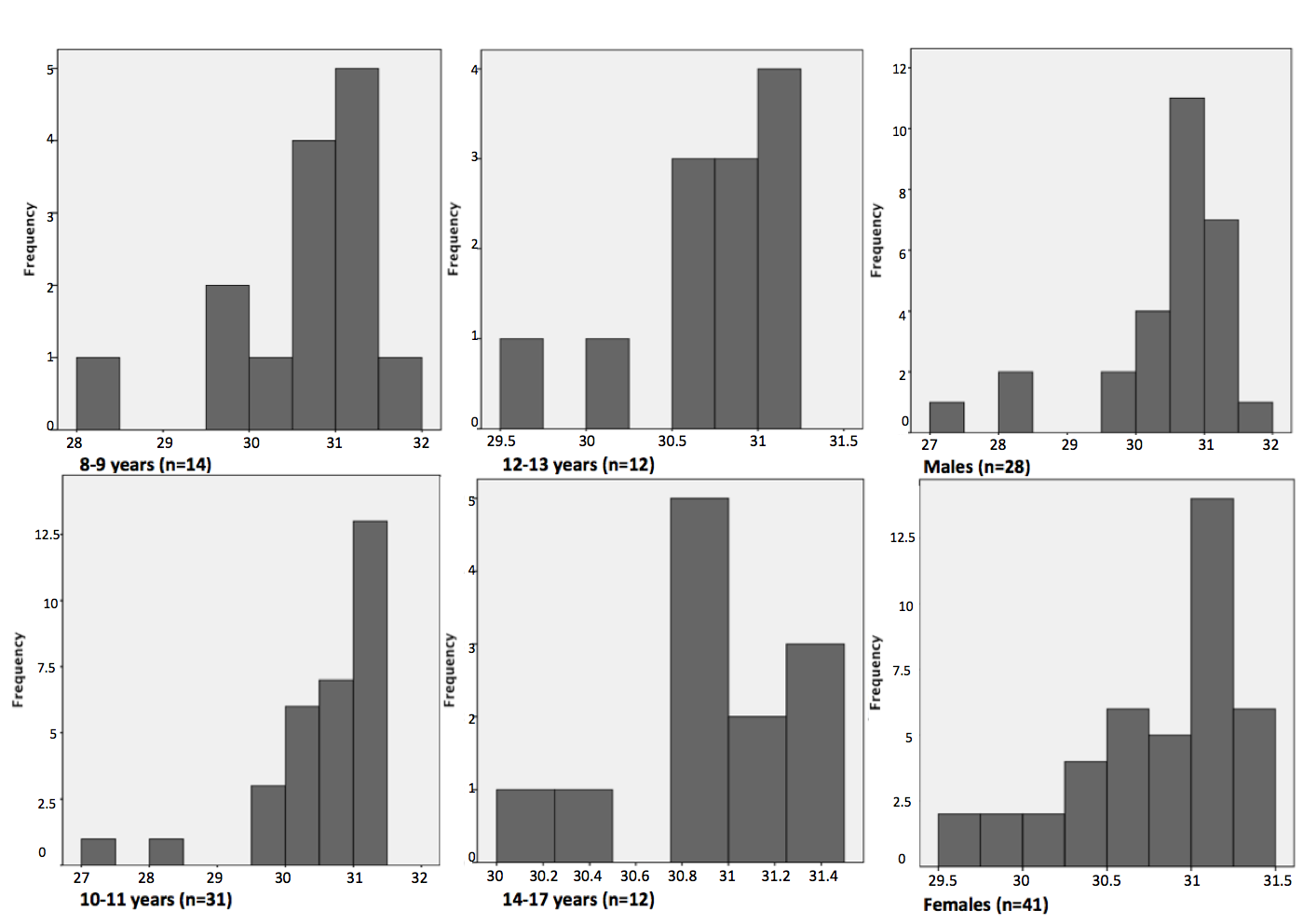

Supplement: Additional file 1: Figure S4 — a - Cold detection threshold. Histograms per subgroup. b - Warm detection threshold. Histograms per subgroup. c - Cold pain threshold. Histograms per subgroup. d - Heat pain threshold. Histograms per subgroup. (ZIP 329 kb) [file 12887_2017_827_MOESM1_ESM.zip › FIGURE_S4A.png]

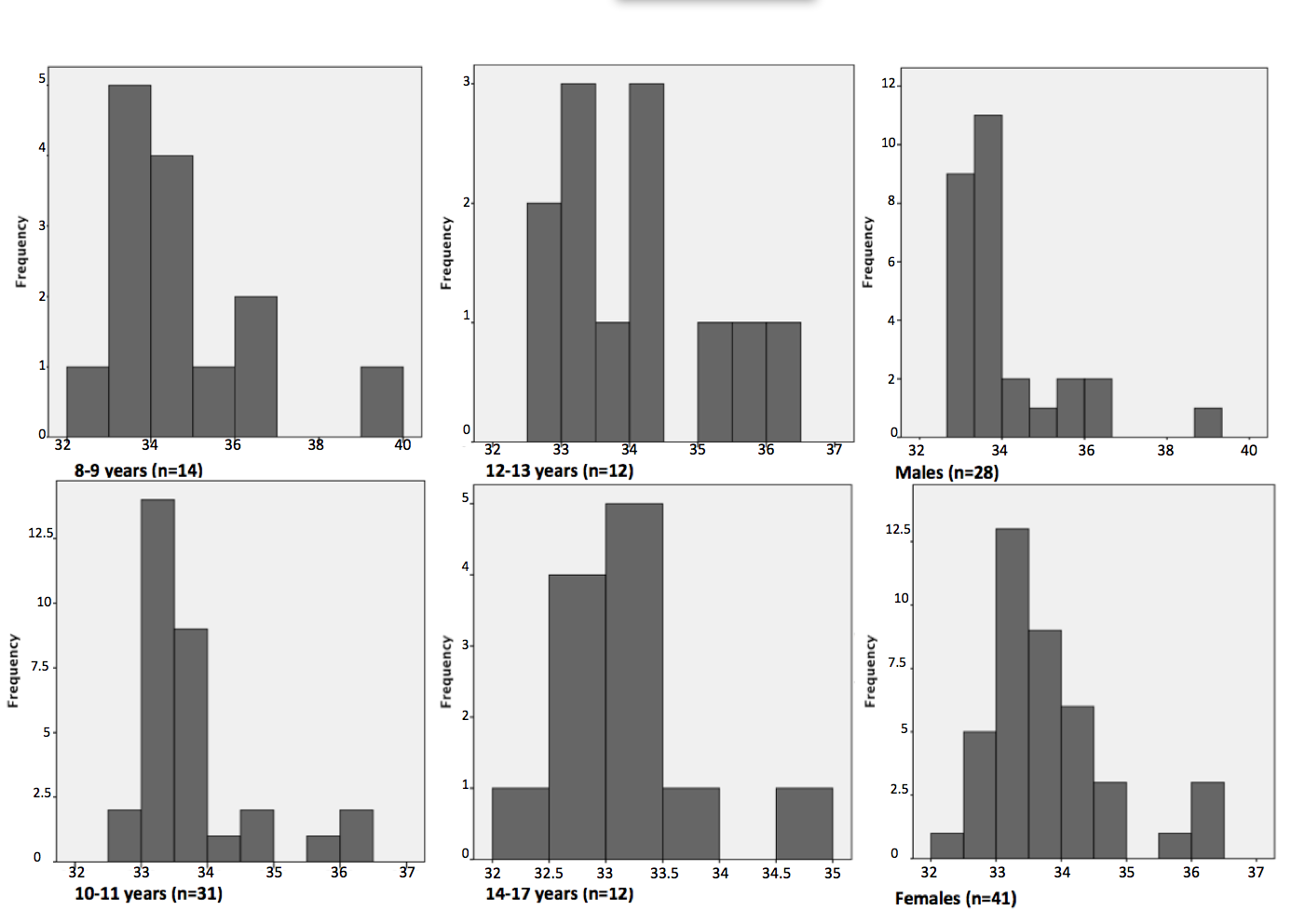

Supplement: Additional file 1: Figure S4 — a - Cold detection threshold. Histograms per subgroup. b - Warm detection threshold. Histograms per subgroup. c - Cold pain threshold. Histograms per subgroup. d - Heat pain threshold. Histograms per subgroup. (ZIP 329 kb) [file 12887_2017_827_MOESM1_ESM.zip › FIGURE_S4B.png]

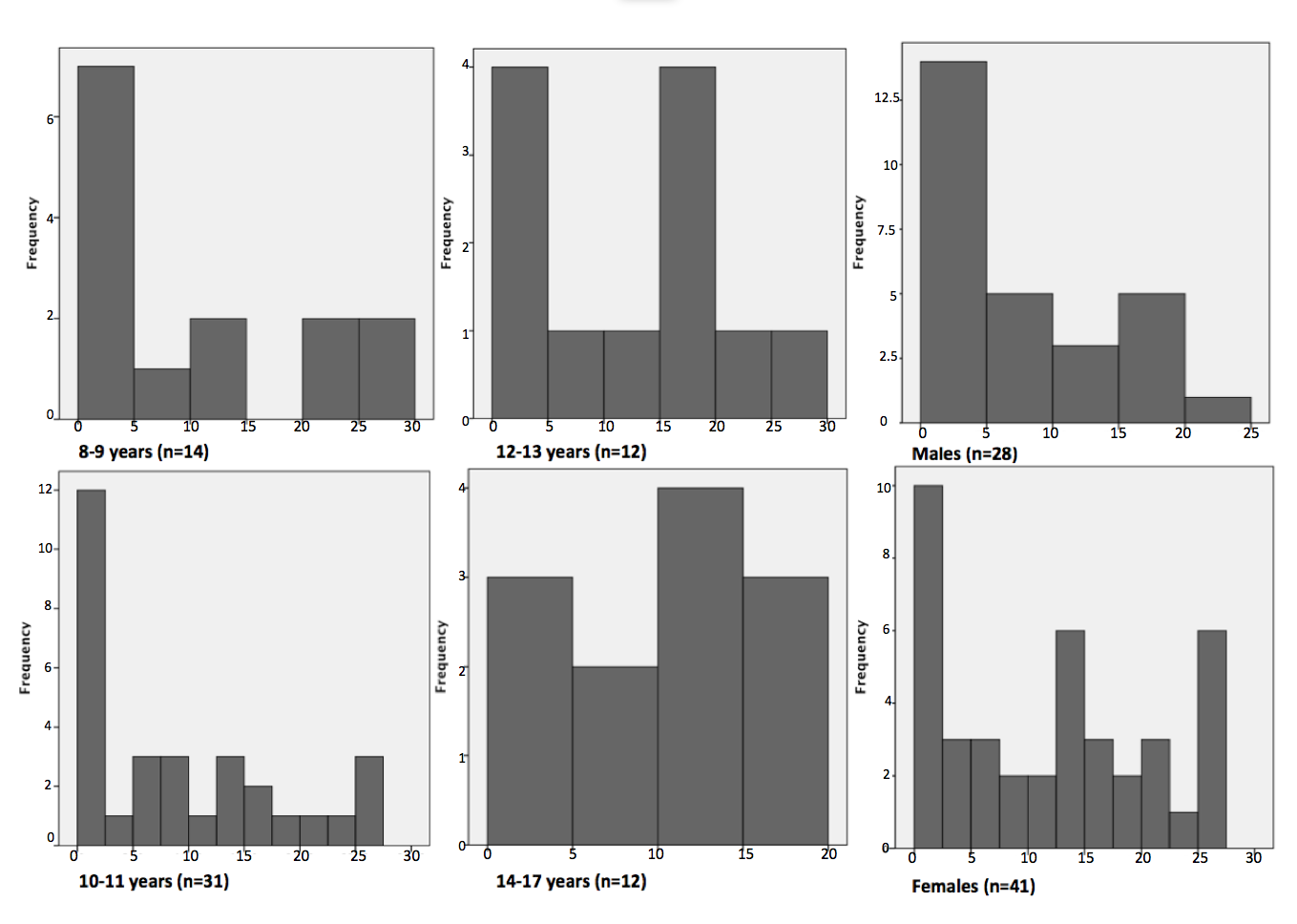

Supplement: Additional file 1: Figure S4 — a - Cold detection threshold. Histograms per subgroup. b - Warm detection threshold. Histograms per subgroup. c - Cold pain threshold. Histograms per subgroup. d - Heat pain threshold. Histograms per subgroup. (ZIP 329 kb) [file 12887_2017_827_MOESM1_ESM.zip › FIGURE_S4C.png]

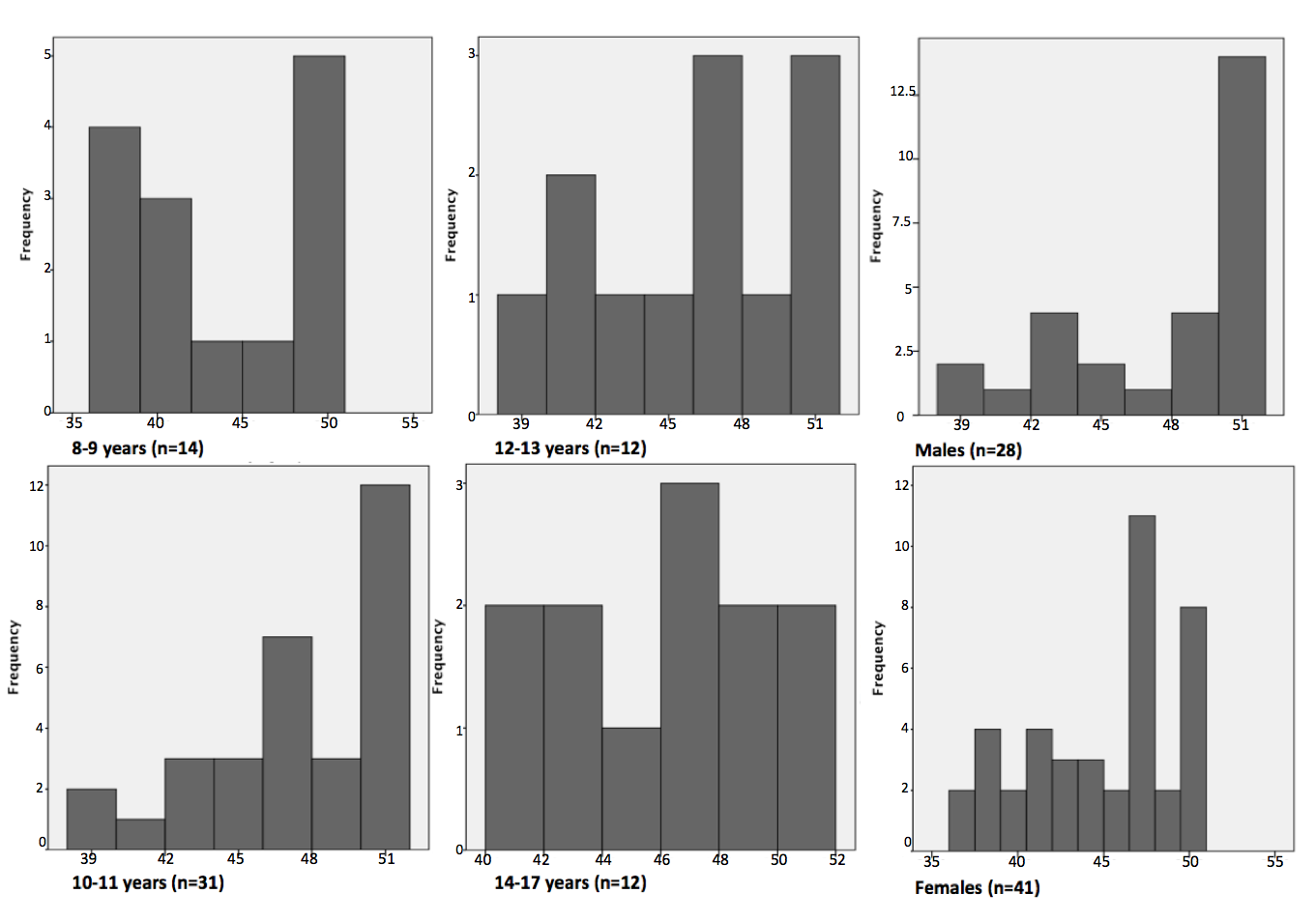

Supplement: Additional file 1: Figure S4 — a - Cold detection threshold. Histograms per subgroup. b - Warm detection threshold. Histograms per subgroup. c - Cold pain threshold. Histograms per subgroup. d - Heat pain threshold. Histograms per subgroup. (ZIP 329 kb) [file 12887_2017_827_MOESM1_ESM.zip › FIGURE_S4D.png]
